# Transcranial focused ultrasound-mediated unbinding of phenytoin from plasma proteins for suppression of chronic temporal lobe epilepsy in a rodent model

**DOI:** 10.1038/s41598-023-31383-4

**Published:** 2023-03-13

**Authors:** Evgenii Kim, Hyun-Chul Kim, Jared Van Reet, Mark Böhlke, Seung-Schik Yoo, Wonhye Lee

**Affiliations:** 1 Department of Radiology, Brigham and Women’s Hospital, Harvard Medical School, 75 Francis Street, Boston, MA 02115 USA; 2 Massachusetts College of Pharmacy and Health Sciences University, Boston, MA USA; 3 Department of Artificial Intelligence, Kyungpook National University, Daegu, South Korea

**Keywords:** Preclinical research, Epilepsy, Drug delivery, Biomedical engineering, Techniques and instrumentation, Pharmacokinetics

## Abstract

The efficacy of many anti-epileptic drugs, including phenytoin (PHT), is reduced by plasma protein binding (PPB) that sequesters therapeutically active drug molecules within the bloodstream. An increase in systemic dose elevates the risk of drug side effects, which demands an alternative technique to increase the unbound concentration of PHT in a region-specific manner. We present a low-intensity focused ultrasound (FUS) technique that locally enhances the efficacy of PHT by transiently disrupting its binding to albumin. We first identified the acoustic parameters that yielded the highest PHT unbinding from albumin among evaluated parameter sets using equilibrium dialysis. Then, rats with chronic mesial temporal lobe epilepsy (mTLE) received four sessions of PHT injection, each followed by 30 min of FUS delivered to the ictal region, across 2 weeks. Two additional groups of mTLE rats underwent the same procedure, but without receiving PHT or FUS. Assessment of electrographic seizure activities revealed that FUS accompanying administration of PHT effectively reduced the number and mean duration of ictal events compared to other conditions, without damaging brain tissue or the blood–brain barrier. Our results demonstrated that the FUS technique enhanced the anti-epileptic efficacy of PHT in a chronic mTLE rodent model by region-specific PPB disruption.

## Introduction

Epilepsy is one of the most common neurological disorders, characterized by recurrent seizures originating from uncontrolled and synchronous neural hyper-excitability^1^. Epilepsy may seriously limit the quality of life for patients and can cause inadvertent accidents associated with seizure events^2^. Based on the location of ictal onset, seizures are typically classified as two major groups—generalized seizures (which affect both sides of the brain) or focal seizures. Mesial temporal lobe epilepsy (mTLE) is a major form of focal epilepsy and a leading cause of intractable epilepsy in adults^3^. Although invasive approaches, such as surgical resection of the ictal locus or electrical deep brain stimulation, have shown promising efficacy in treating recalcitrant/intractable epilepsy^4^, pharmacological intervention is still considered the frontline treatment.

Among available choices of anti-epileptic agents (e.g., valproic acid or carbamazepine), phenytoin (PHT) has been a widely prescribed anti-epileptic drug, enlisted in the essential medicines list (EML, by the World Health Organization) as a major anti-convulsant medication^5^. PHT, administered orally or through intravenous (*i.v.*) injection, binds to and blocks voltage-gated sodium channels, leading to suppression of seizure occurrences with preventive utility against recurrent seizure events of focal onset and life-threatening status epilepticus (SE)^6^.

PHT has a high binding affinity (~ 90%) for plasma proteins, primarily to albumin^7^. The PHT-albumin complex in the blood does not cross the blood–brain barrier (BBB) due to the large molecular weight (MW) of albumin (66.5 kDa), limiting its pharmacological action. Only the unbound portion of PHT (MW: 252 Da) can cross the BBB into the brain parenchyma. An increase in the systemic dose would increase the concentration of therapeutically effective PHT but can lead to serious side effects, including hepatic necrosis, ataxia, and psychological alterations (e.g., suicidal thoughts)^8^. Thus, the development of an alternative strategy to regionally increase PHT concentration in the central nervous system (CNS) has been sought after.

The force governing plasma protein binding (PPB) is known to be mediated by non-covalent bonds (hydrogen bonds^9^, van der Waals forces, and hydrophobic effects^10^) at the molecular level^11,12^, and is relatively weak (~ 10^–12^ N) and rapidly reversible. Recent investigations have demonstrated that the radiation force generated by acoustic pressure waves of ultrasound may overcome the binding force between plasma proteins and drugs within biological tissues. Therefore, ultrasound application offers a new means to non-invasively increase the effective (unbound) concentration of a drug^13–15^. For example, sonication given at 1 MHz enhanced the effects of finasteride (which binds to albumin) on promoting hair growth in a murine model of androgenic alopecia^14^, and 500 kHz ultrasound was used to promote local anesthetic effects of lidocaine by unbinding it from alpha-1 acid glycoprotein^15^, another major constituent of plasma proteins.

When delivered in a focal manner (e.g., using an acoustic lens, the shape of a transducer, or phased-arrays), acoustic pressure waves can be applied to a specific region-of-interest on the order of a few millimeters in diameter, imparting either thermal or mechanical energy to the tissue^16^. The technique is called focused ultrasound (FUS) and has been used in various applications, including tissue ablation (e.g., ablation of uterine fibroid) and lithotripsy^17^. Low-frequency range (typically < 1 MHz) ultrasound pressure waves, due to a longer wavelength than the waves used in diagnostic imaging applications (on the order of 2–15 MHz), can be transcranially transmitted to a localized brain area through an intact skull. The transcranial FUS technique has been utilized to ablate region-specific brain tissue using high acoustic intensities^18^ and has started to offer novel neurotherapeutic brain stimulation options at low-intensity (without damaging the sonicated neural tissues)^19^.

Based on the ability to reach deep brain regions with exquisite spatial selectivity, we have previously shown that the application of non-thermal low-intensity FUS to a brain hemisphere in rodents increased parenchymal uptake of PHT without altering the BBB integrity, through unbinding the drug from plasma albumin^13^. We were motivated to evaluate the preclinical utility of the technique to enhance the anti-epileptic effects of PHT by applying pulsed FUS to the ictal focus among a rodent mTLE model, created by intrahippocampal injection of kainic acid (KA) to the unilateral hippocampus. As sonication parameters (i.e., the choice of pulse duration [PD] and pulse repetition frequency [PRF]) may affect unbinding efficiency, the effects of different combinations of sonication parameters on unbinding PHT from albumin were first evaluated using equilibrium dialysis prior to the animal study. Upon determining a set of sonication parameters that yielded the maximum unbinding, FUS was administered, in multi-day sessions, to a group of rats having chronic mTLE that were receiving PHT. The degree of electrographic seizure activities was evaluated across the different experimental conditions (i.e., including the groups that received only PHT or FUS). The safety of the technique was assessed by histology of brain samples obtained immediately after and one month after the sonication sessions.

## Methods

### FUS transducer

**Figure 1:**
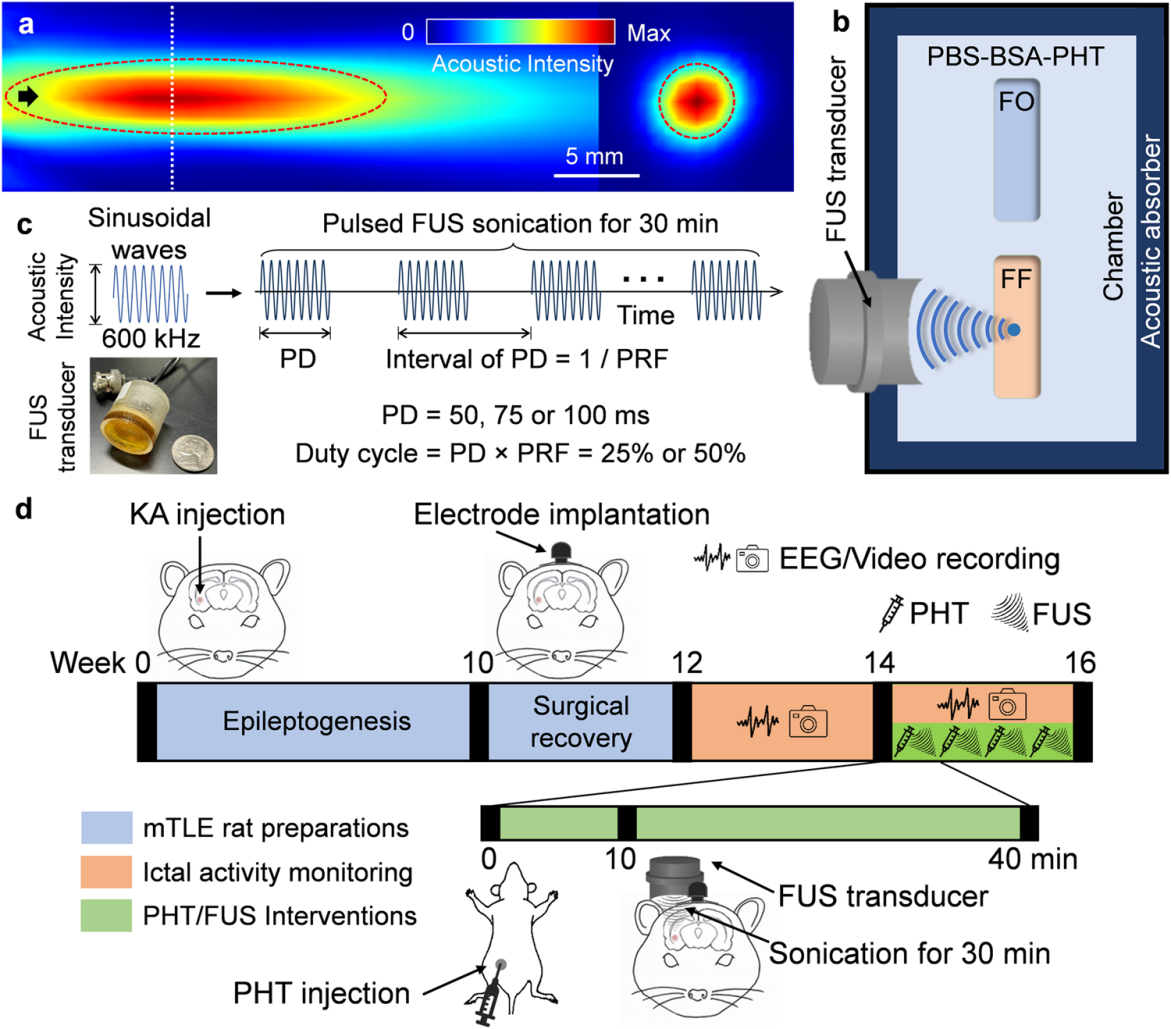
Schematics of the equilibrium dialysis setup and the animal experiments. (**a**) Ultrasound beam profile of the in-house built FUS transducer in acoustic intensity (W/cm^2^). The intensity map on the longitudinal plane (left) was measured 10 mm away from the exit plane of the transducer while the transverse profile (right) was measured at the acoustic focus (located 20 mm away from the exit plane), indicated as a white dotted line. The FWHM intensity profile is depicted with dotted red lines. The black arrow indicates the direction of sonication. (**b**) Illustration of the equilibrium dialysis settings, depicting the dialysis chamber containing PBS-BSA-PHT cocktail solution, a FUS transducer mounted to the chamber wall, and two submerged dialysis cassettes–one at the acoustic focus (‘FF’), the other outside the path of sonication (‘FO’). Rubber pads at the chamber inner walls were used as acoustic absorber. (**c**) The acoustic parameters used. Inset: the in-house built FUS transducer. (**d**) Diagram of the animal experiments with a timeline of 16 weeks. The mTLE rat models were prepared with intrahippocampal KA injection and electrode head pedestal implantation (weeks 0–12, in blue). EEG ictal activity monitoring sessions with video recording were performed in post-KA weeks 12–16 (in orange). PHT and/or FUS interventions were administered four times in weeks 14–16 (in green), along with EEG/video recording sessions. The EEG data pair of the 2-week period before (weeks 12–14; pre-intervention) and during (weeks 14–16; post-intervention) the treatments were compared to examine the seizure-suppressive effects. *BSA* bovine serum albumin, *EEG* electroencephalogram, *FUS* focused ultrasound, *FWHM* full-width at half-maximum, *mTLE* mesial temporal lobe epilepsy, *PBS* phosphate-buffered saline, *PD* pulse duration, *PHT* phenytoin, *PRF* pulse repetition frequency.

A miniature single-element FUS transducer (26.5 mm in diameter and 26.5 mm in height, weighing 19 g) was built in-house to deliver sonication to the epileptic focus, without interfering with the implanted electrode head pedestal (see “Overview of experiments involving rodent mTLE model” in “Methods”). The ultrasound beam, generated by a lead zirconate titanate (PZT) ceramic disc with a diameter of 19.1 mm (600 kHz center frequency, APC International Ltd., Mackeyville, PA), was focused by a plano-concave polyetherimide acoustic lens having a 20 mm radius of curvature (Armset LLC, Middleton, MA) abutted to the piezoceramic disc. The housing for the transducer was 3D-printed (Form 3, Formlabs, Somerville, MA). The transducer was actuated with pulsed sinusoidal waves (600 kHz fundamental frequency) from a function generator (33210A, Keysight, Santa Rosa, CA) that were amplified using a 10 W linear amplifier (Sonomo 500, Electronics and Innovation Ltd, Rochester, NY). The acoustic intensity at the focus was measured and calibrated to the input voltage amplitudes using a hydrophone (HNR0500, Onda Corporation, Sunnyvale, CA). The spatial profile of the acoustic field was mapped in degassed water using a needle-type hydrophone (HNC200, Onda Corporation) mounted to a 3-axis mechanical stage (BiSlide, Velmex Inc., Ontario County, NY), covering a 31 × 31 mm^2^ transversal plane and a 51 × 31 mm^2^ longitudinal plane along the sonication axis, using methods described previously^20^. The acoustic focus was formed 20 mm away from the exit plane of the transducer. The size of the focus was 5 mm in diameter and 22 mm in length, at the full-width at half-maximum (FWHM) of the intensity profile (Fig. 1a). The transducer could generate up to ~ 10 W/cm^2^ of spatial-peak pulse-average intensity (I_SPPA_) at the focus.

### In vitro equilibrium dialysis

A 3D-printed chamber was used to perform the equilibrium dialysis experiments to examine the effects of FUS on unbinding PHT from bovine serum albumin (BSA, A2153, MilliporeSigma, Burlington, MA). PHT (PHR1492, MilliporeSigma) and BSA were dissolved in phosphate-buffered saline (PBS, pH 7.4, Gibco 10010, Thermo Fisher Scientific, Waltham, MA) at concentrations of 15 µg/mL and 45 mg/mL, respectively, that mimic actual physiological conditions in humans^21^. A 200 mL volume of the PBS-BSA-PHT solution was poured into the chamber (Fig. 1b). Two dialysis cassettes (7-kDa cutoff pore size; Slide-A-Lyzer, Thermo Fisher Scientific) filled with PBS (without PHT/BSA) were placed in the slots within the chamber—one at the FUS focus (noted as ‘FF’), and the other outside the sonication path (‘FO’). The inner walls of the chamber were lined with ~ 5-mm thick rubber pads to absorb the sound waves. A 7-kDa cutoff dialysis membrane of the cassette allows for the diffusion of unbound, free PHT (252 Da) into the cassette while blocking the BSA-PHT complex (~ 69 kDa). The experiments were conducted with the solution equilibrated to ambient room temperature (~ 20–24 °C).

FUS was administered to the middle of the dialysis window of cassette ‘FF’ for 30 min with a fixed I_SPPA_ of 5 W/cm^2^. The corresponding peak rarefactional pressure (P_r_) was 0.38 MPa, with a mechanical index (MI) of 0.49, much lower than the safety guideline limit of 1.9 MI for soft tissue sonication set by the U.S. Food and Drug Administration (FDA)^22^. A total of 6 different sets of sonication pulsing parameters were tested, with a combination of two duty cycles (DCs = 25% and 50%) and three PDs (50, 75, and 100 ms) along with PRFs (Supplementary Table S1; the parameter illustrated in Fig. 1c). The pulsing parameters were chosen based on our previous study^13^ and were different from those that induce suppressive neuromodulatory effects (i.e., the use of DC of ~ 5% and PD of ~ 0.5 ms)^23,24^. The use of 25% and 50% DCs were examined based on the postulation that higher acoustic energy deposition would lead to higher unbinding efficiency, and corresponded to spatial-peak temporal-average intensities (I_SPTA_) of 1.25 and 2.50 W/cm^2^, lower than an upper limit of 3 W/cm^2^ I_SPTA_ for therapeutic equipment set by the International Electrotechnical Commission (IEC) 60601 part 2 standard^22^. Ten measurements were taken for each parameter set, including a control condition (without sonication, referred to as ‘Ctrl’). The temperature of the chamber bath/solution was monitored every 5 min in a contactless fashion using a calibrated infrared thermal camera (sensitivity of ~ 0.5 °C; C3, FLIR Systems Inc., Wilsonville, OR). The dialysates recovered from the cassettes were analyzed for unbound PHT concentrations using high-performance liquid chromatography (HPLC). Details of the HPLC method are described elsewhere^13^.

### Overview of experiments involving rodent mTLE model

The schematics of animal experiments are illustrated in Fig. 1d. The mTLE rodent model was prepared using a unilateral intrahippocampal injection of KA to male Sprague–Dawley rats (~ 270–280 g, n = 30; Charles River Laboratories, Wilmington, MA). After showing classic signs of short-term SE by the KA injection, the animals developed spontaneous recurrent seizures (SRS) after a few weeks of epileptogenesis, which plateaued ~ 12–15 weeks after the KA injection^25^. Around 10 weeks post-KA injection, we surgically implanted an electrode head pedestal mounted to the anterior part of the skull to allow for plug-in/-out of a wireless electroencephalogram (EEG) transmitter (EPTX-10214, Biopac Systems Inc., Goleta, CA). After waiting ~ 2 weeks to allow for wound healing from the surgery, we monitored for the presence of seizures for 2 weeks (3 h/day, 4 days/week, a total of 24 h). EEG was also acquired, synchronized with video recording, which characterized the mTLE SRS activities before interventions (pre-intervention period).

During post-KA weeks 14–16, the epileptic rats were randomly divided into three groups (n = 10 in each group) with different interventions of receiving: (1) both PHT and sonication (PHT^+^/FUS^+^), (2) PHT-only (PHT_ONLY_), and (3) sonication-only (FUS_ONLY_), all of which were performed under anesthesia with *i.p.* injection of ketamine/xylazine. PHT (75 mg/kg, Dilantin injectable, West-Ward Pharmaceuticals Co., Eatontown, NJ) was injected intraperitoneally (*i.p.*) and FUS was stereotactically administered to the epileptic focus around the KA injection site through the intact skull (~ 10 min after PHT injection in PHT^+^/FUS^+^ group). The interventions were administered 4 times with a time gap of 3–4 days for 2 weeks across all groups, and on-site EEG/video recordings were performed 3 h/day for 2 consecutive days after the treatment (generating a total of 24 h EEG/video data during the post-intervention period). The time intervals between the treatment and EEG/video recording sessions were either 24 h or 48 h (per animal). We also examined the effects of repeated PHT^+^/FUS^+^ sessions using a separate group of male non-epileptic rats (without KA injection, body weight = 452 ± 11 g, n = 3; balanced with post-KA week 12 mTLE rats’ body weight of 447 ± 32 g) through the same EEG/behavioral monitoring. All epileptic rats were sacrificed immediately after the completion of seizure activity monitoring (i.e., after 16 weeks of scheduled experiments) for brain harvest.

### Intrahippocampal kainic acid injection

All animal experiments were conducted under the approval of the Institutional Animal Care and Use Committee (IACUC) of Brigham and Women’s Hospital (protocol 2020N000034). The reporting in this study is in compliance with the ARRIVE guidelines (https://arriveguidelines.org/). Animals were cared for in accordance with the guidelines set forth by the IACUC and the Guide for the Care and Use of Laboratory Animals^26^. The animals’ vivarium was maintained with controlled environment (12 h light/dark cycles with lights on 7 a.m.–7 p.m., ~ 23 °C, ~ 50% relative humidity), with access to feed and water ad libitum.

The chronic focal mTLE model was prepared using intrahippocampal injection of KA (Tocris Bioscience, Bristol, UK), based on previous literature^25,27^. The surgical procedure was performed under ketamine/xylazine anesthesia (80:10 mg/kg, i.p.). The fur over the scalp was removed with an electric clipper and depilatory cream (Nair, Church & Dwight Co., Inc., Ewing Township, NJ). Then, the rats were placed on a stereotactic frame (ASI Instruments, Warren, MI) after the application of ophthalmic ointment to the eyes. An incision was made along the midline of the scalp, and the skull was exposed to identify the location of the bregma. Topical anesthesia (lidocaine hydrochloride injectable 2%, Vedco Inc., Saint Joseph, MO) was applied over the skull. A burr hole was then drilled using a micro drill burr (19008-07, Fine Science Tools Inc., Foster City, CA) over the injection site (5.6 mm caudal and 4 mm right to the bregma), leaving the dura intact. A 30G needle (7803-07, Hamilton Company, Reno, NV) attached to a 10 μL gastight syringe (1701 RN, 7653-01, Hamilton Company) was inserted through the burr hole in the calvarium, so that it reached the targeted brain area in the right dentate gyrus—CA1/CA3 (AL: − 5.6 mm, ML: 4.0 mm, DV: 7.0 mm to the bregma). KA was injected with a dose of 1 μg/μL (in normal saline) at a rate of 1 μL/min for 1 min using a microinjection syringe pump (Legato^®^ 130, KD Scientific Inc., Holliston, MA). After injection, the needle was left in place for an additional 5 min and was slowly retracted to allow for the tissue to close shut. Once the needle was removed, the burr hole was filled with bone wax (W31G, Ethicon Inc., Raritan, NJ), and the incision site was sutured. During ~ 4–10 h post-op, the rats were monitored for seizure activities, including SE. If the rat showed self-injurious behaviors, such as biting or wild running/jumping, the animal was gently restrained with a wooden stick until it was stabilized. The body weight and the occurrence of epileptic behaviors (e.g., SE) were monitored 2 times daily during the first week of post-op and at least every other day during the period of mTLE SRS development.

### Electrode head pedestal implantation

A wireless EEG setup was chosen over the typical tethering configuration to minimize the confounding effects from animal stress caused by tangled wires^28^. A 3-channel electrode head pedestal (diameter = 3.5 mm, height = 8 mm, wire diameter/length = 0.127 mm/7 mm, MS333/3-A/SPC, P1 technologies, Roanoke, VA) was surgically implanted over the skull, offering a plug-in and plug-out connection with a telemetry EEG transmitter. The pedestal implantation was performed under ketamine/xylazine anesthesia (80:10 mg/kg, *i.p.*), ten weeks after KA injection. After exposing the skull, three small skull-mounted anchor screws (without penetrating the cranium) were placed around the pedestal’s base, located ~ 3 mm rostral to the bregma in the midline, to provide mechanical stability to the implantation^29^. The location of the pedestal was chosen not to block the sonication path to the KA injection site (i.e., the epileptic focus in the brain). The two recording wires from the electrode were placed bilaterally above the parietal bones, while the common/reference electrode wire was placed near the nasal bone. The base of the pedestal, along with the anchor screws, was fixed to the skull by applying cyanoacrylate medical adhesive (Loctite Prism 4541, Henkel, Düsseldorf, Germany). While exposing the top portion of the pedestal, the scalp incision was sutured back around the pedestal base, and the electrode was capped (303DC, Dust cap, P1 technologies). The animals were then singly-housed to prevent the accidental detachment of the electrode head pedestal when socially-housed.

### EEG/video recording with on-site monitoring of seizure activity

EEG acquisition with video recording was performed for 3 h per session, within a time window of 9 a.m.–6 p.m. considering circadian rhythm^25^, to monitor spontaneous seizures and corresponding EEG ictal activities from chronic mTLE rats. The assignments of 3 h-long EEG acquisition time windows (in either the morning or afternoon) were randomized and balanced across the three experimental groups. The wireless EEG transmitter that contains a built-in pre-amplifier (with a gain of 800) and radio transmission circuits^30^ (foot print = 8 × 13 mm^2^, height = 29 mm, weight = 2.5 g, 2 channels, EPTX-10214, Biopac Systems Inc.) was plugged into the implanted electrode after light sedation through ~ 2–4 min exposure to 3–4% isoflurane (also applied during the removal of the transmitter from the pedestal). The EEG transmitter was turned off when not in use (Epoch-ACTI, Biopac Systems Inc.).

The rat with the EEG transmitter was moved to a transparent cage, placed on top of an EEG receiver tray (Epoch2, Biopac System Inc.) that was located within and grounded to a Faraday shield for noise reduction. EEG signal recorded from the hemisphere ipsilateral to the KA-injection site was further digitized (PowerLab 4/35 or 8/30 and LabChart Pro v.7.3.8 software, ADInstruments, Colorado Springs, CO) at a sampling rate of 10 kHz. Animal behavior was also video-recorded and displayed on the same LCD screen, in sync with the EEG, by a stream webcam (Logitech C922 Pro HD, Lausanne, Switzerland) using a module software in LabChart Pro (Video capture v.1.1.4, ADInstruments). Animal behavior and the raw EEG signal, along with 30-Hz low-pass filtered EEG time series, were monitored on-site and noted regarding the timing and behaviors that were potentially related to the mTLE spontaneous seizures.

### Screening and characterization of spontaneous seizures and ictal activities

Based on the notes during on-site monitoring, the behavioral/EEG seizure activities (‘ictal events’) were screened offline by two independent reviewers replaying the synchronized EEG/video recording. The seizure characteristics (type of ictal events, duration, and behavioral manifestation) were determined based on the consensus of three independent reviewers. The EEG time series data (48 h for each animal) were reviewed on each time window of 10 s to document ictal events that were not noted during the on-site monitoring. To distinguish the ictal activities from physiological noise or movement artifacts, the EEG signal features (e.g., repetitive sharp EEG waves in a range of 2–20 Hz having the amplitudes that deviate from inter-ictal duration) that last at least several seconds according to well-documented criteria are considered ictal events^25^. Since head-shaking, bruxing, and boggling behaviors could also yield repetitive sharp EEG wave changes from non-epileptic rats^31^, we defined the minimum duration of 3 s to be classified as an ictal event (as confirmed by separate measurements from three non-epileptic rats, results not shown). Each ictal event was also scored with video reviews on the basis of a modified Racine’s scale^25,32^ categorization of 1–5: facial movements (scale 1), head nodding/frozen behavior (scale 2), clonus (scale 3), rearing (scale 4), and rearing and falling (scale 5). The time information (e.g., start and end of the ictal events) was tagged to the EEG data for subsequent numerical analysis.

### Data analysis of the chronic TLE-related EEG ictal activities

**Figure 2:**
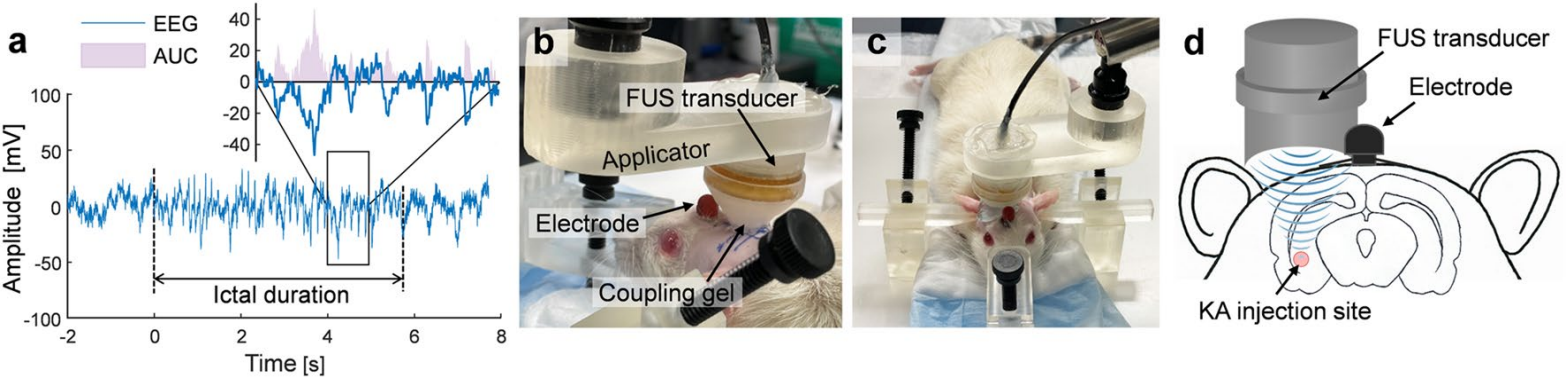
An exemplar ictal EEG segment and the transcranial FUS sonication setup. (**a**) Illustration of the derivation of ictal duration and ictal amplitude from an exemplar EEG segment. The dotted lines indicate the beginning and end points of the ictal event with repetitive sharp EEG waves, defining the ictal duration. The ictal amplitude was derived as the area under the curve of the absolute values of EEG signals (AUC, in pink) divided by the ictal duration. Inset: a magnified view of the selected square area. (**b–d**) Transcranial FUS sonication setup viewed from the (**b**) left behind and (**c**) front of the mTLE rat. The FUS transducer connected to a swivel/lockable applicator was stereotactically placed over the head, posterior to the implanted electrode head pedestal, for the sonication targeting to the KA injection site. An acoustic coupling gel was inserted between the transducer and scalp. (**d**) Illustration of FUS sonication delivery to the KA injection site.

The labeled EEG data were analyzed in Matlab (ver. R2021b, MathWorks, Natick, MA). The time-averaged EEG amplitude for each ictal event (namely ‘ictal amplitude’) was calculated by area under the curve (AUC) divided by the ictal duration (an example shown in Fig. 2a). Then, the following ‘seizure indices’ were derived per each animal from pre- and post-intervention periods separately (each 24 h): (1) count of ictal events (‘ictal count’), (2) mean of ictal durations ('ictal duration/event'), and (3) mean of ictal amplitudes (‘ictal amplitude/event’). These ‘seizure indices’ were compared between the pre- and the post-intervention periods to examine the presence of differential effects on seizure suppression across the three experimental groups (i.e., PHT^+^/FUS^+^, PHT_ONLY_, and FUS_ONLY_).

For the group-level analysis, normalized ‘seizure indices’ were derived for each animal by post-intervention ‘seizure indices’ divided by pre-intervention ‘seizure indices.’ With the normalized ‘seizure indices,’ one-way analysis of variance (ANOVA) followed by the Tukey–Kramer post-hoc analysis with Bonferroni correction was used to compare the indices across the three experimental groups. The significance level of *p* < 0.05 was set for statistical analysis, unless otherwise noted. All statistical tests are also listed in the text and figure legends. All data were represented as mean and standard deviation, unless specified.

### Transcranial application of FUS to the epileptic focus

Among the experimental groups that received sonication (PHT^+^/FUS^+^ and FUS_ONLY_ conditions), FUS was transcranially delivered to the epileptic focus while the animals were anesthetized with *i.p.* injection of ketamine/xylazine. The fur over the scalp was removed as necessary before the sonication. Then, the rats (either after or without PHT injection depending on the experimental condition) were placed on a custom-built stereotactic frame (Fig. 2b,c). The FUS transducer was attached to an articulated arm (Zacuto, Chicago, IL), utilized to adjust and lock the location and orientation of the transducer to align the acoustic focus to the epileptic focus. A compressible polyvinyl alcohol (PVA, 341584, MilliporeSigma) hydrogel, prepared in a shape of a conical frustum (with rounded bottom) through two freeze–thaw cycles (detailed methods described previously^33^), was inserted between the scalp and FUS transducer for acoustic coupling. A generic ultrasound hydrogel (Aquasonic 100, Parker Laboratories Inc., Fairfield, NJ) was applied to all interfaces on the sonication path. In the case of PHT^+^/FUS^+^ condition, 30 min of sonication was administered to the site of KA injection ~ 10 min after PHT injection (depicted in Fig. 2d), using the selected parameters (see “Evaluation of sonication parameters through equilibrium dialyses” in “Results”) of 50 ms PD at 50% DC with 10 Hz PRF at 5 W/cm^2^ I_SPPA_. The temperature of the scalp around the FUS entry point was measured before and after sonication via the infrared camera (C3, FLIR; having 0.5 °C sensitivity which was calibrated to 30 °C non-reflecting object using a thermistor). Upon completion of FUS session and recovery from anesthesia, the animals were returned to the vivarium.

### Post-intervention assessments of brain histology and BBB integrity

The effects of repeated FUS and/or PHT interventions to the brain tissue were examined using histological assessments. Within 2 days of completion of the last EEG/video monitoring session, the mTLE rats were anesthetized with ketamine/xylazine *i.p.* and sacrificed via transcardial perfusion using normal saline and 10% formalin solution (SF100-20, Thermo Fisher Scientific). The skull was extracted and immersion-fixated in formalin solution overnight. The brains were harvested, and the coronal sections were collected to contain the site of KA injection. The presence of necrosis or neuronal loss, ischemic neurons, glial infiltration and degenerated neurons, or apoptosis was examined through hematoxylin and eosin (H&E), vanadium acid fuchsin (VAF)-toluidine blue, glial fibrillary acidic protein (GFAP) immunohistochemistry (IHC), and caspase-3 IHC, respectively. Considering the inherently impaired brain of the mTLE rats, the safety of the technique was also examined on non-epileptic normal rats that underwent the same PHT^+^/FUS^+^ sessions and were sacrificed 2 days (n = 3) or 1 month (n = 2) after the last sonication, with the same set of histological and IHC analyses. Among a few mTLE rats (n = 9), the presence of reactive astrocytes/microglia, as a typical manifestation of hippocampal sclerosis^25^, was examined with vimentin IHC and CD11b IHC to assess the presence of any differences on these IHCs across the different experimental groups as well as to confirm the proper preparations of the epileptic model. For the animals that received PHT, PHT IHC was assessed to examine the distribution of residual PHT in the brain parenchyma (a few days) after the multiple PHT^+^/FUS^+^ or PHT_ONLY_ sessions. To examine the post-FUS BBB integrity, tail vein injection of 4% trypan blue (872.9 Da, 302643, MilliporeSigma) in normal saline was performed to epileptic (n = 2) and non-epileptic (n = 1) rats immediately after the last sonication (PHT^+^/FUS^+^) session, and the animals were sacrificed after ~ 1 h systemic circulation of the trypan blue.

## Results

### Evaluation of sonication parameters through equilibrium dialyses

**Figure 3:**
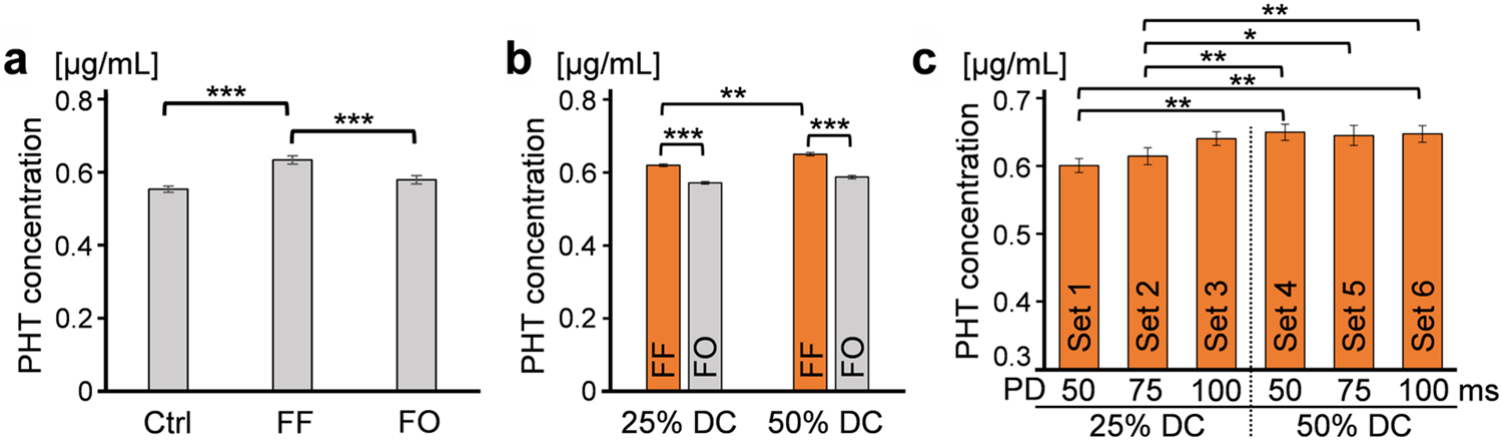
Unbound, free PHT concentrations in dialysates following equilibrium dialysis of PBS-BSA-PHT solution with different conditions. The PHT levels were compared (**a**) among the samples of unsonicated control (‘Ctrl’), at the FUS focus (‘FF’), and outside the focus (‘FO’), with one-way repeated measures ANOVA (***p < 0.001), (**b**) between the samples with 25% and 50% DC conditions, with two-tailed paired *t*-test (**p < 0.01, ***p < 0.001), and (**c**) across the ‘FF’ samples with the varying sonication parameter sets 1–6 in Supplementary Table S1, with one-way repeated measures ANOVA (*p < 0.05, **p < 0.01). Data shown as mean ± standard error.

The PHT concentrations were compared among the dialysate samples recovered from the control condition (‘Ctrl’), the sonicated cassettes at the FUS focus (‘FF’) with the varying sonication parameters (i.e., all samples across all tested pulsing schemes), and the cassettes outside ultrasound path (‘FO’) (Fig. 3a). One-way repeated measures ANOVA (*F*(2,14) = 38.7, *p* < 0.001) with Bonferroni adjustment showed a significantly higher concentration of unbound PHT level from ‘FF’ compared to those from ‘Ctrl’ and ‘FO’ (both *p* < 0.001, with ~ 14% and ~ 10% relative increases, respectively); however, no significant difference was observed between ‘Ctrl’ and ‘FO’ (*p* > 0.1). When the effects of DC were compared, 50% DC yielded a higher PHT concentration than 25% DC (*p* < 0.01, paired *t*-test, two-tailed, n = 23; Fig. 3b). In the comparison among the six sets of FUS parameters (Supplementary Table S1 and Figs. 1c, 3c), the highest concentration was achieved from the use of 50 ms PD with 10 Hz PRF (parameter set 4), which yielded an unbound PHT level of 0.65 ± 0.10 μg/mL at ‘FF’ (~ 18% increase compared to ‘Ctrl’), whereas ‘Ctrl’ and ‘FO’ showed 0.55 ± 0.07 μg/mL and 0.59 ± 0.09 μg/mL, respectively. One-way repeated measures ANOVA (*F*(5,35) = 2.97, *p* < 0.05) with the least significant difference (LSD) test showed that parameter sets 4–6 (50% DC) yielded significantly higher levels of PHT unbinding compared to those from parameter sets 1 and 2 (all *p* < 0.05, Fig. 3c), which were confirmed by the pairwise comparison (Fig. 3c, p < 0.05, paired *t*-test, two-tailed, n = 8). Although the differences among parameter sets 4–6 were statistically marginal, parameter set 4 (50 ms PD with 10 Hz PRF) that yielded the highest unbound PHT level was chosen to be used in the subsequent in vivo animal studies.

### Suppression of electrographic ictal activity

**Figure 4:**
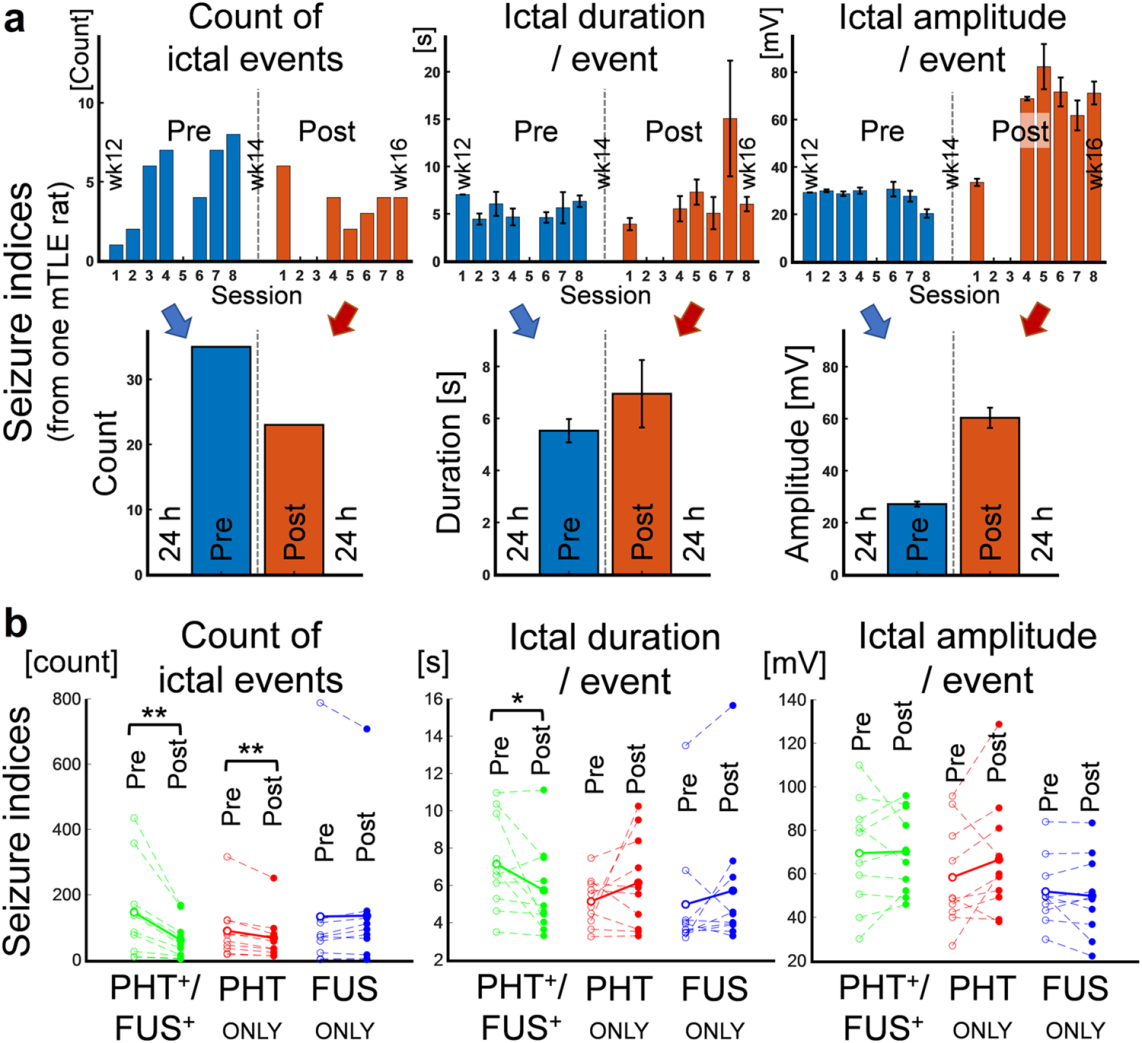
The change of the three seizure indices before (Pre) and during/after (Post) interventions. (**a**) 3 h-long EEG session-by-session data (upper) and 24 h pre-/post-data (lower) from the 8 EEG sessions (3 h-long each) of an exemplar mTLE rat with PHT_ONLY_ intervention. (**b**) 24 h pre-/post-data across the PHT^+^/FUS^+^ (green), PHT_ONLY_ (red), and FUS_ONLY_ (blue) groups (n = 10 rats/group). The dashed lines link the pre- (empty circles) and post-intervention (filled circles) values from each individual rat, and the solid lines link the mean values of the pre-/post-intervention seizure indices within each group. *p < 0.05, **p < 0.01 (paired *t*-test, one-tailed).

12 weeks after KA injection, all animals exhibited ictal activities along with seizure behaviors. The three seizure indices of (1) count of ictal events (‘ictal count’), (2) ictal duration/event, and (3) ictal amplitude/event were extracted for each mTLE rat from 24 h EEG/video data before (pre-intervention) and during/after intervention (post-intervention), respectively. An exemplar data set from one mTLE rat with PHT_ONLY_ condition is shown in Fig. 4a, and all of the individual mTLE rats’ seizure indices and the group averaged values are shown in Fig. 4b. The seizure indices were compared between the pre- and post-interventions to examine the differential effects on seizure suppression across the three groups (i.e., PHT^+^/FUS^+^, PHT_ONLY_, and FUS_ONLY_).

The seizure indices during the pre-intervention period were not significantly different among the three experimental groups (one-way ANOVA with Tukey–Kramer post-hoc analysis, *F*(2,27) = 0.31, *F*(2,27) = 2.00, *F*(2,27) = 1.17, with all *p* > 0.1 for count of ictal events, ictal duration/event, ictal amplitude/event, respectively). The comparison showed a significant reduction in the number of ictal events during PHT_ONLY_ and PHT^+^/FUS^+^ treatments (from 89 ± 88 events to 67 ± 70 events, and from 145 ± 145 events to 61 ± 61 events, respectively; paired *t*-test, one-tailed, both *p* < 0.01), but not in the FUS_ONLY_ interventions (from 133 ± 233 events to 135 ± 207 events, *p* = 0.5). The ictal duration/event was significantly decreased in the PHT^+^/FUS^+^ group (from 6.9 ± 2.3 s to 5.7 ± 2.4 s; paired *t*-test, one-tailed, *p* < 0.05), while it was marginally increased for the PHT_ONLY_ and FUS_ONLY_ conditions (from 5.1 ± 1.3 s to 6.1 ± 2.5 s, and from 5.0 ± 3.1 s to 5.7 ± 3.7 s, respectively). The ictal amplitude/event was not significantly changed by any of the interventions (paired *t*-test, one-tailed, all *p* > 0.1).

**Figure 5:**
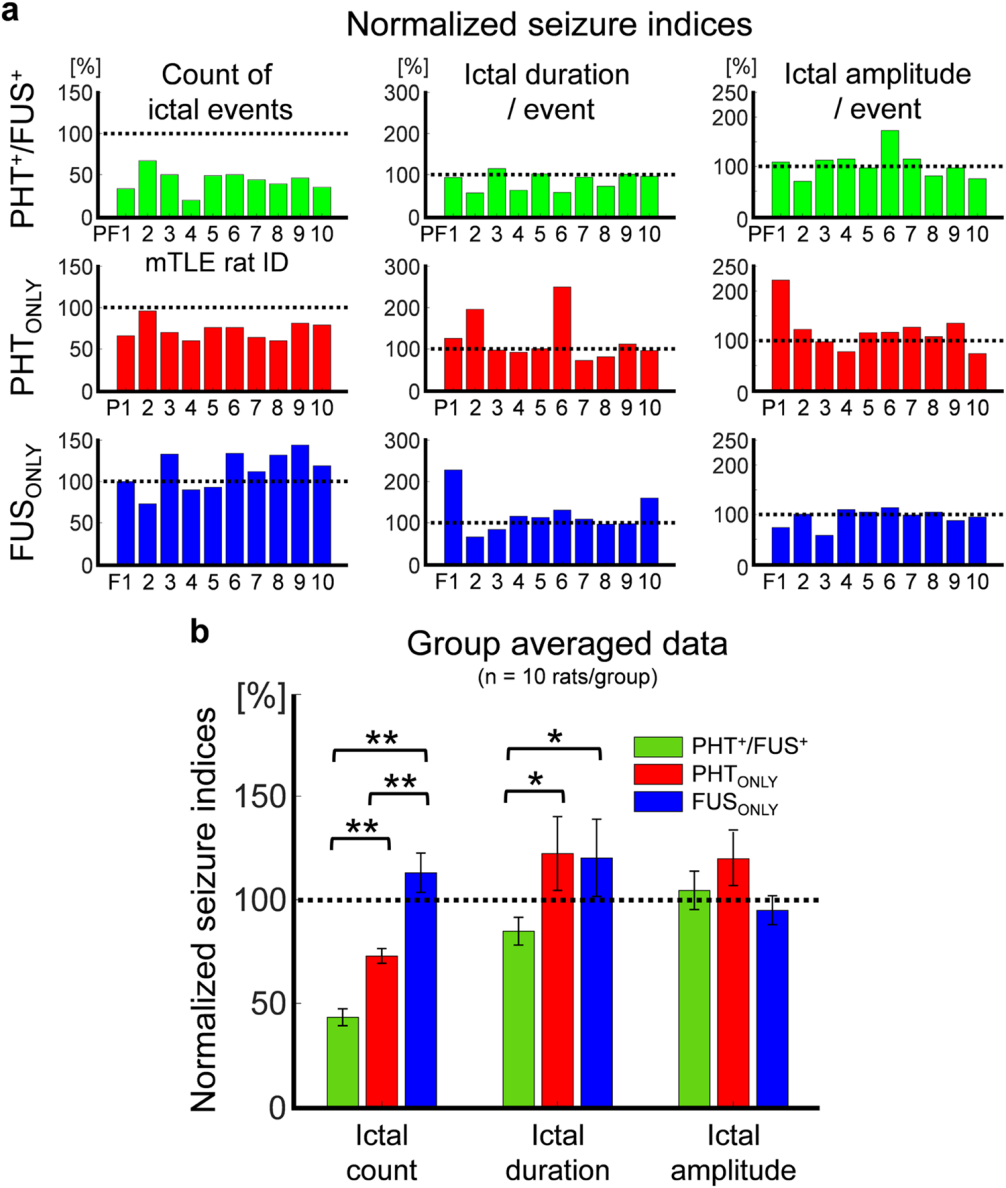
Illustrations of the normalized seizure indices. The post-intervention seizure indices (count of ictal events, ictal duration/event, and ictal amplitude/event) were normalized to pre-intervention seizure indices from the PHT^+^/FUS^+^ (green), PHT_ONLY_ (red), and FUS_ONLY_ (blue) groups (n = 10 rats/group). (**a**) Individual rat’s normalized data from Fig. 4b. (**b**) Group averaged normalized seizure indices from (**a**), shown as mean ± standard error. *p < 0.05 (*t*-test, one-tailed), **p < 0.01 (one-way ANOVA with Tukey–Kramer post-hoc analysis).

Considering the mTLE rats’ individual variabilities in terms of ictal activities as shown in Fig. 4b, the seizure indices obtained during intervention sessions (data points with ‘filled circle’ in Fig. 4b) were normalized with respect to the seizure indices obtained during the pre-intervention period (data points with ‘empty circle’ in Fig. 4b). The normalized seizure indices for each individual mTLE rat (with IDs of 'PF1–10', 'P1–10’, and 'F1–10’ for respective PHT^+^/FUS^+^, PHT_ONLY_, and FUS_ONLY_ groups) are shown in Fig. 5a and the group averaged results are shown in Fig. 5b. The normalized ‘ictal count’ was significantly different among the three groups (one-way ANOVA, *F*(2,27) = 44.2, *p* < 0.001, followed by a Tukey–Kramer post-hoc analysis with all *p* < 0.01). While the number of ictal events increased for rats that received FUS_ONLY_ (13% ± 24%), it decreased for the animals under PHT_ONLY_ interventions (− 27% ± 11%). The number of ictal events was further reduced when the rats were treated by PHT with FUS (PHT^+^/FUS^+^, − 57% ± 13%). In the case of normalized ‘ictal duration/event,’ one-way ANOVA did not show any significant differences among groups (*F*(2,27) = 2.32, *p* = 0.1), but the multiple *t*-test results showed a significant decrease for rats under PHT^+^/FUS^+^ interventions (decrease by 15% ± 21%), compared to PHT_ONLY_ and FUS_ONLY_ groups (increase by 22% ± 57%, 20% ± 46%; one-tailed *t*-test, *p* = 0.03 and *p* = 0.02, respectively). The normalized ‘ictal amplitude/event’ did not show any statistical differences among the three treatment groups (one-way ANOVA, *F*(2,27) = 1.66, *p* = 0.2).

### Evaluation of seizure reduction based on a modified Racine’s scale

**Figure 6:**
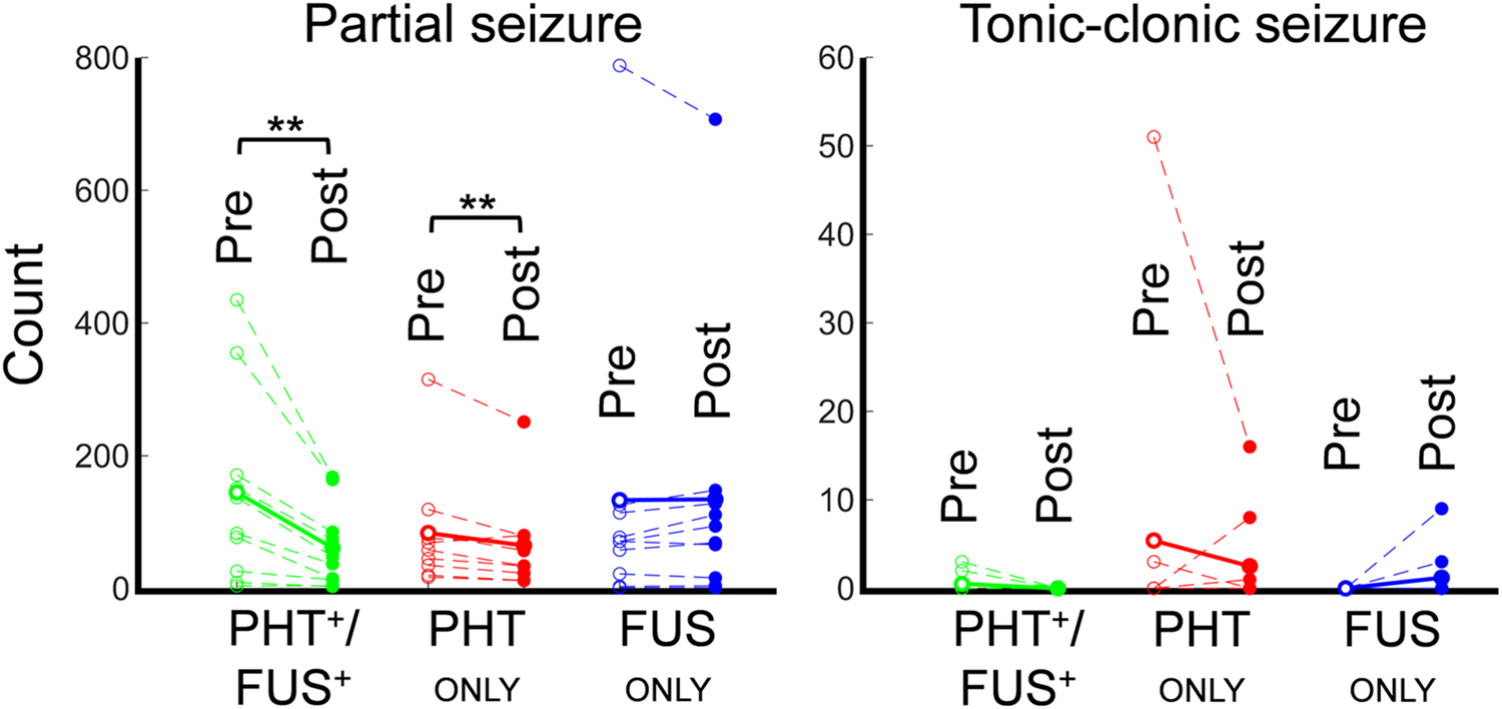
Changes of behavioral seizure activities based on the modified Racine’s scale. The number of partial and tonic–clonic seizure events appeared during pre- (empty circles) and post- (filled circles) intervention periods from PHT^+^/FUS^+^ (green), PHT_ONLY_ (red) and FUS_ONLY_ (blue) groups (n = 10 rats/group). The dashed lines link the pre- and post-intervention values from each individual rat, and the solid lines link the mean values of the pre-/post-intervention ictal counts within each group. In the right panel, the data from mTLE rats that did not show tonic–clonic seizures are overlapped as a single dashed line on the x-axis for each experimental group, respectively. **p < 0.01 (paired *t*-test, one-tailed).

The ictal events were classified into two groups based on the modified Racine’s scale: a partial seizure (scale 1 and 2), and a tonic–clonic seizure (scale 3–5). The number of seizure events corresponding to their types is shown in Fig. 6. A significant reduction of partial seizure count was observed in PHT_ONLY_ and PHT^+^/FUS^+^ treatments (from 83.6 ± 86.9 events to 64.8 ± 70.2 events and from 144.7 ± 145.4 events to 61.1 ± 62.0 events, respectively; paired *t*-test, one-tailed, both *p* < 0.01), however, there was no change from the FUS_ONLY_ intervention (from 133.1 ± 233.9 events to 134.4 ± 207.6 events,* p* = 0.5). Although a small number of animals showed tonic–clonic seizure events (2, 2, and 0 rats before and 0, 3, and 2 rats after PHT^+^/FUS^+^, PHT_ONLY_ and FUS_ONLY_, respectively), the marginal decrease in their occurrences after the interventions was also observed in PHT_ONLY_ and PHT^+^/FUS^+^ groups (from 5.4 ± 16.0 events to 2.5 ± 5.3 events and from 0.5 ± 1.0 events to 0 event, respectively), whereas FUS_ONLY_ showed an increased trend (from 0 event to 1.2 ± 2.9 events).

### Assessment of BBB disruption

**Figure 7:**
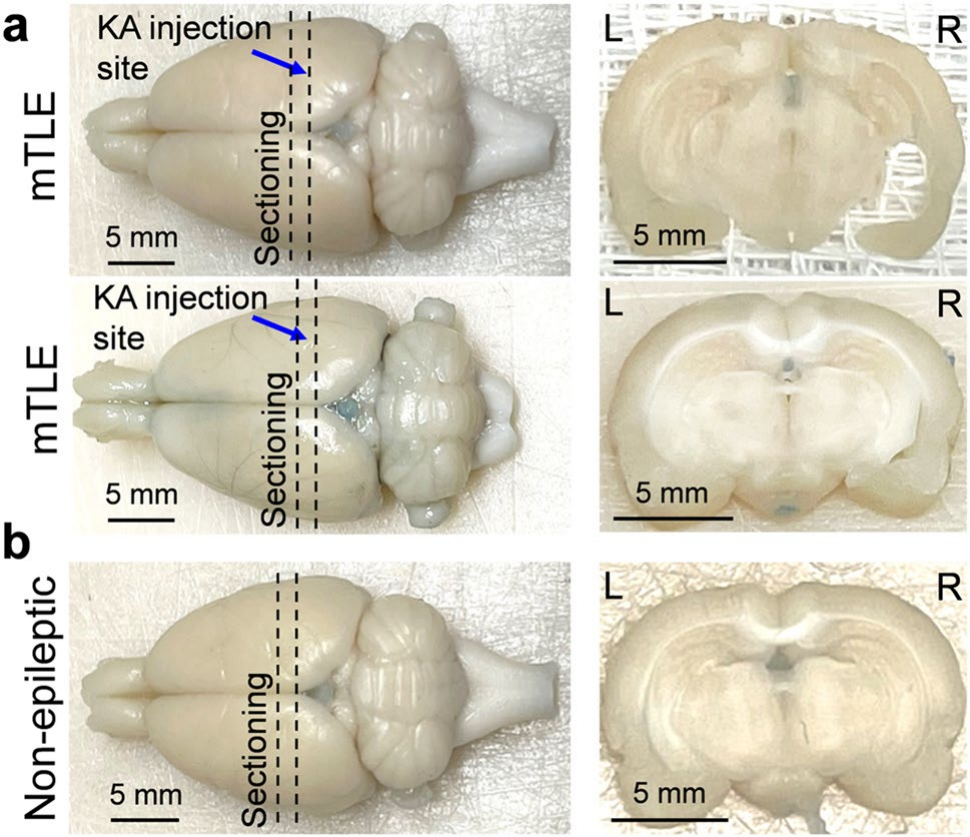
Assessment of post-FUS BBB integrity. Tail vein injection of trypan blue immediately after PHT^+^/FUS^+^ treatments revealed no signs of BBB disruption in brain specimens from (**a**) two mTLE rats and (**b**) a non-epileptic rat. The blue arrow indicates the location of KA injection site and dotted lines illustrate the sectioning locations. *L* left, *R* right.

Visual examinations of the gross brain specimens and their coronal sections from the mTLE rats (n = 2, Fig. 7a) and a non-epileptic rat (n = 1, Fig. 7b) that underwent the tail vein injection of trypan blue immediately after PHT^+^/FUS^+^ treatments did not reveal any visible signs of extravasation of the dye, indicating the absence of post-FUS BBB disruption.

### Safety profile with thermal, behavioral, and histological assessments

The scalp temperature measurement right after sonication did not show any thermal increase above the animal body temperature (35.9 ± 1.2 °C, 34.8 ± 1.6 °C, before and after sonication, respectively; paired *t*-test, one-tailed, *p* = 0.99). All the non-epileptic control animals (without KA injection) showed normal behavior and health status after the multiple sessions of the same PHT^+^/FUS^+^ treatments that were administered to mTLE rats. In the case of the epileptic animals, other than the chronic mTLE-related status (such as SRS), further behavioral exacerbations were not observed after the interventions of PHT^+^/FUS^+^, PHT_ONLY_, or FUS_ONLY_.

**Figure 8:**
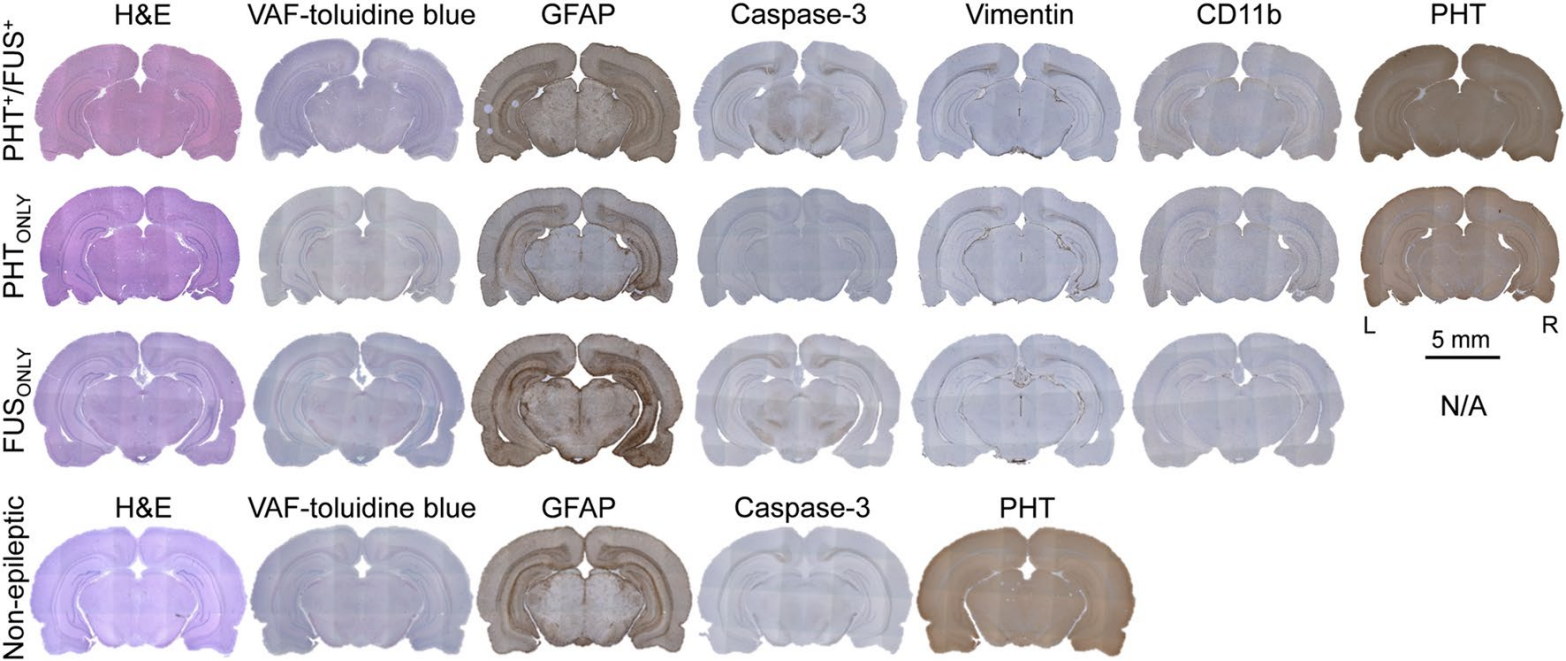
Representative histology and IHC results. These slides were obtained from mTLE rats of PHT^+^/FUS^+^ (top), PHT_ONLY_ (2nd row) and FUS_ONLY_ (3rd row) groups as well as a non-epileptic rat (bottom) that underwent the same PHT^+^/FUS^+^ treatments. IHC slides of PHT for FUS_ONLY_ condition and vimentin and CD11b for non-epileptic rats are not shown as they are not applicable (N/A). In the PHT^+^/FUS^+^ condition, the three white dots on the left side of GFAP IHC were staining artifacts. *L* left, *R* right.

Exemplar histological data from KA-injected, epileptic rats are shown in Fig. 8 (top to 3rd rows). Anticipated unilateral hippocampal atrophy, ipsilateral to the KA injection site, was observed in the H&E stain obtained from most of the animals. The tissue dorsal to the injection site, which was exposed to the sonication path, did not show any signs of damage through H&E, VAF and caspase-3 IHC. Glial infiltrations (GFAP IHC) and the presence of reactive astrocytes (Vimentin IHC and CD11b IHC) were also prevalent in the site of KA injection but were not detected at the sonication pathway. The PHT IHC did not reveal any specific increase in absorption at the sonicated site, which suggests that the presence of a region-specific increase in PHT uptake might have been balanced between the hemispheres (or distributed across the brain tissue) by the time that the animal underwent the necropsy (~ 2 days after the completion of the sonication). In the case of non-epileptic control rats, the histological examinations (H&E, VAF-toluidine blue, GFAP, and caspase-3 staining) performed 2 days and a month after the last PHT^+^/FUS^+^ session showed no apparent signs of cell or vascular damage (Fig. 8, bottom row).

## Discussion

Enhancement of targeted drug delivery to the brain is challenging, especially without disrupting the integrity of the BBB. In the present study, we applied low-intensity transcranial FUS to the epileptic focus in chronic mTLE rats that received the anti-epileptic drug PHT and examined whether unbinding PHT from plasma proteins can boost its anti-epileptic efficacy. The application of FUS enhanced the suppression of electrographic and behavioral seizures without damaging the sonicated brain tissues and without increasing the systemic dose or disrupting the BBB.

Prior to in vivo animal studies, the effects of acoustic parameters (DCs and PDs) on efficiency of PHT unbinding (from albumin) were explored at 5 W/cm^2^ I_SPPA_ using in vitro equilibrium dialysis. Between the two different DCs tested (Fig. 3b), the use of 50% DC (I_SPTA_ of 2.50 W/cm^2^) yielded significantly higher levels of unbound PHT compared to the use of 25% DC (I_SPTA_ of 1.25 W/cm^2^). This indicates that a higher acoustic energy deposition per given time (i.e., I_SPTA_) offered a superior unbinding efficacy. Based on this observation, the use of continuous waves (i.e., 100% DC) could be investigated to enhance the unbinding efficacy; however, it may accompany elevated risks for heat generation at the acoustic focus. Although the use of 50 ms PD yielded a marginal increase in unbound PHT level compared to those from other PD conditions (i.e., parameter sets 1–3 at 25% DC or sets 4–6 at 50% DC; Fig. 3c), 50 ms PD and 10 Hz PRF (set 4) was selected for subsequent in vivo experimentation.

Within-group comparisons of the seizure indices between the pre- and post-intervention periods confirmed the anti-epileptic efficacy of PHT, shown by the decreased ‘number of ictal events’ (Fig. 4b). Application of FUS enhanced the effects of PHT, as evidenced by reductions in ‘ictal duration/event’ and in ‘number of ictal events’. Between-group analysis also showed that concomitant application of both PHT and FUS reduced the normalized ‘ictal count’ and ‘ictal duration/event’ compared to those from administration of PHT alone (Fig. 5b). These within- and between-group comparisons jointly suggest that application of FUS with administration of PHT demonstrated enhanced anti-epileptic efficacy of PHT. It is also interesting to note that pharmacological treatment alone marginally increased ‘ictal duration/event’ and ‘ictal amplitude/event’. This finding is consonant with previous studies on application of PHT among kindled rats^34^ and suggests that the primary impact of PHT is to decrease the risk for the occurrence of seizure episodes, rather than to reduce the severity or duration of ‘active’ seizures.

The presence of individual variabilities in ictal activities was anticipated based on previous studies^25,27^, and we observed considerable variabilities across the chronic mTLE model in terms of the seizure indices (Fig. 4b). As to the cause for the variability, we conjecture that several intrinsic or extrinsic factors, such as individual differences in KA injection site, effects from anesthesia^27^, the onset of SRS periods^35^, and circadian rhythm^25^, might have contributed. Despite the presence of individual variabilities, with randomized and balanced assignments of 3 h-long EEG acquisition time windows (in either the morning or afternoon) across the experimental groups, the seizure indices acquired during the pre-intervention period were not statistically different across the experimental groups, indicating that between-group variabilities before treatment did not skew the results (Fig. 5).

In terms of behavioral seizure activities, the number of partial seizures was reduced by PHT, which was further enhanced by concurrent FUS administration (Fig. 6). Although the number of animals that manifested tonic–clonic seizure activities (during the EEG/video recording period) was small, it is interesting to note that none of the animals showed the modified Racine’s scale 3–5 seizures during the period of PHT treatments combined with FUS, whereas a number of rats (2–3 out of 10 animals in each group) showed tonic–clonic seizures during the interventions of PHT only or FUS alone. These results support the capability of FUS to enhance the therapeutic efficacy of PHT on the suppression of behavioral seizures, which agreed with reductions of the electrographic ictal activities.

We note that the application of FUS is known to suppress neuronal excitability, as demonstrated by suppression of (1) visual evoked potentials/blood oxygenation level-dependent (BOLD) functional magnetic resonance imaging (fMRI) signals in small animals^20,23^ and (2) cortical and thalamic motor activity in ovines^24,36^. However, the administration of FUS alone did not exhibit suppressive effects on the seizure activities (Figs. 4, 5, 6). Although the utility of suppressive neuromodulatory potentials of FUS on anti-epileptic applications started to emerge^37–43^, we conjecture that the sonication parameters used in the present research (e.g., 50 ms PD, 50% DC), which were vastly different from studies utilizing much shorter PDs (e.g., 0.3–1 ms) that have been adopted to suppress regional brain tissue excitability, were only effective in selectively unbinding PHT from plasma proteins without showing neuromodulatory effects.

In the assessments of PHT IHC, there was no localized increase in PHT uptake around the sonicated brain tissue, whereas a FUS-mediated region-specific increase in PHT uptake was observed in our previous study^13^. We surmised that the timing of the brain harvest attributed to the discrepancies. While the previous PHT IHC was performed on the brains harvested immediately after the completion of the FUS along with PHT *i.p.* injection^13^, the brains were harvested ~ 2 days after the last sonication due to the EEG/video recording sessions. During the waiting period, the increased region-specific PHT uptake with FUS sonication might have been redistributed across the brain parenchyma indistinctively due to the long elimination time (on the order of hours)^44^. Thus, a prolonged application of the PHT unbinding using FUS may be beneficial to maintain the regionally increased PHT uptake for an extended duration.

As to the potential mechanisms behind the FUS-mediated drug unbinding from plasma albumin, thermal effects or cavitation (which needs a pressure of 13.5 MPa or higher^45^) are ruled out due to the use of low-intensity pulsed ultrasound (P_r_ of 0.38 MPa, I_SPPA_ of 5 W/cm^2^ with 50% DC, MI of 0.49). Considering ~ 17% acoustic pressure attenuation through the rat skull/scalp with ~ 600 kHz FUS^46^, in situ P_r_ at the FUS target in the brain was estimated as 0.32 MPa. As PPB is known to be primarily governed by relatively weak (~ 10^–12^ N) and transient non-covalent bonding^9–12^, we, therefore, provide a simplified estimate of acoustic radiation force imposed on the PHT-albumin complex using the information of acoustic pressure (in situ P_r_ = 320 kPa = 3.2 × 10^5^ N/m^2^) and the surface area (SA) of the PHT-albumin complex. Assuming the shape of albumin is an ellipsoid with a ~ 14 nm long axis and a ~ 3 nm short axis^47^, the SA of albumin is estimated to be ~ 105.11 × 10^–18^ m^2^ (the size of PHT is not considered due to its small MW of 252 Da). Presuming that half of the SA is exposed to the in situ P_r_ with 20% acoustic absorption, the acoustic radiation force applied on the PHT-albumin complex is 3.4 × 10^–12^ N (20% of in situ P_r_ × SA/2 = 0.2 × [3.2 × 10^5^ N/m^2^] × [52.55 × 10^–18^ m^2^]), which is much stronger than the binding force. Based on this estimation, we surmise that the acoustic radiation force is a plausible mechanism behind the observed phenomena^13,14^.

The unbinding and rebinding of PHT to plasma proteins or brain proteins/lipids would occur constantly. In the in vitro equilibrium dialysis settings, unbound PHT can freely diffuse in and out through the dialysis membranes even during active sonication. In the case of brain parenchyma, on the other hand, we surmise that a portion of unbound PHT via FUS, once passing the BBB, may rapidly rebind to brain proteins/lipids (due to high lipid solubility) and remain in the parenchyma^48^ (until being cleared from the brain). In terms of the membrane area close to unbound PHT via FUS, the total area of multiple endothelial membranes within the acoustic focal volume (3D prolate spheroid) would be much larger than that of the two in vitro dialysis membranes. It is noteworthy that, in our previous study^13^, unbound PHT level increased 27% with 55 min FUS compared to unsonicated samples in in vitro equilibrium dialysis, while PHT IHC after 35 min FUS showed 190% higher PHT level in the sonicated region than unsonicated areas in the rat brain. The enhanced in vivo effects of FUS-mediated PHT unbinding may explain the dramatic decreases in ictal count (~ 30%) and ictal duration (~ 40%) with the present FUS technique compared to the PHT only condition (Fig. 5b).

Regarding the safety profile of the interventions, the behavioral and histological assessments of the epileptic rats did not reveal any evidence of further exacerbations of the mTLE manifestations (e.g., SRS) or cellular/vascular tissue damage due to the FUS sonication (Fig. 8). The non-epileptic rats’ behavioral and histological assessments confirmed the acute and long-term safety after the multiple FUS sessions. Temperature changes can also affect the degree of PPB to drugs^49^; however, the present use of a low intensity (2.5 W/cm^2^ I_SPTA_), far below the threshold for thermal effects^50^, did not elevate the skin temperature at the sonication entry. The absence of temperature elevations during the equilibrium dialysis, being supported by the tissue phantom studies and numerical acoustic simulations in our previous report^13^, suggests that thermal effects of FUS were not likely involved. During its clinical translation, more advanced in vivo temperature monitoring, such as magnetic resonance-based thermometry^51^ can be used.

Trypan blue *i.v.* injection immediately after the application of FUS confirmed that the given FUS parameters did not disrupt the BBB (Fig. 7). It is noteworthy that the present FUS technique was performed in the absence of microbubble contrast agents (MBs). FUS-MB-mediated disruption of the BBB, with a pressure below the threshold of eliciting tissue damage, has been shown to locally deliver large MW pharmacological agents^52,53^, and has thus been implicated in the delivery of chemotherapeutic agents (e.g., doxorubicin^54^) or neurotransmitters (e.g., GABA^55^). However, it accompanies a potential risk of hemorrhaging and exposure to external pathogens when excessive acoustic intensity is delivered^56^. The present technique, however, does not use MBs to open the BBB, thus providing a novel means of delivering CNS drugs that have a high rate of PPB. Such pharmacological agents include other anti-epileptic drugs (e.g., valproic acid or carbamazepine), medications to treat cognitive symptoms (e.g., donepezil) and anti-depressants (e.g., fluoxetine or paroxetine), and constitute subjects for future investigations on enhancing their delivery using the proposed technique. Other than the use for CNS drugs, potential applications of the technique can be sought for non-CNS drugs that strongly bind to plasma proteins. For instance, cisplatin, a chemotherapy drug with a PPB rate of 98% that is commonly used in the treatment of testicular or cervical cancer^57^, can be a candidate drug to examine the technique’s capability for promoting region-specific therapeutic effects. When targeting tumors, the use of thermal FUS with a higher intensity may also be considered to achieve a greater drug unbinding efficacy as well as additional anti-tumor effects induced by hyperthermia^58^.

Other than unbinding drugs from plasma proteins, the proposed FUS technique may be used to reduce the aggregation of harmful endogenous macro protein molecules. For example, neuroactive oligomers or higher order assemblies (e.g., beta-sheets, associated with Alzheimer’s disease) are formed by beta-amyloid (Aβ) monomers via the van der Waals and electrostatic interactions^59^. Monoclonal antibody-based drugs (e.g., trastuzumab) tend to aggregate (by non-covalent interactions) during the manufacturing/storage periods, not only reducing drug efficacy but also inciting undesired immunogenic responses^60^. As these binding mechanisms are similar to those of the PPB to drugs, further investigations are warranted to examine if FUS-mediated unbinding techniques are capable of disrupting such aggregations.

We acknowledge the limitations of the current study. First, the systemic or localized serum level of PHT was not measured, although we believe that the proposed method would not affect the systemic serum PHT level. The measurement of systemic/localized PHT level using blood sampling, in vivo microdialysis, or PHT IHC before, during, or after the application of FUS following PHT injections will demonstrate its regional effects on unbinding. However, the technical confounders that are associated with in vivo microdialysis, such as the inevitable disruption of the BBB during surgical implantation of the dialysis probe or the considerable time necessary to reach signal equilibration after sonication^61^, need to be properly addressed. Also, investigations of cellular level effects were not conducted, and future studies on examining the post-FUS effects on paracellular or transcellular pathways and BBB permeability would provide more insights regarding the mechanism behind the FUS-mediated drug unbinding technique. In addition, we also recognize the potential confounding factors on ictal activity, being introduced by intermittent FUS sessions in different days^27^ under the anesthesia, which may confound antiepileptic efficacy. This warrants further studies among freely behaving unanesthetized animals with wearable FUS systems^24,28,29^, which would enable more frequent (e.g., daily) sonication sessions and continuous 24 h EEG/behavioral monitoring. Direct recording of field potentials from the ictal hippocampal areas may offer automated and unsupervised analysis of electrographic seizure activities^62^, which will also provide additional information on the effects of circadian rhythm on ictal activities^63^. Quantification of histology and IHC data, such as volume change of hippocampus or the ratio of GFAP positive signal^42^, was not conducted in this study, and further investigations on quantitative analysis of histology/IHC would provide information to further elucidate the in vivo effects of FUS-mediated PHT unbinding technique.

## Conclusions

We have demonstrated that FUS-mediated PHT unbinding from plasma proteins (e.g., albumin) proximal to the epileptic focus resulted in enhanced anti-epileptic efficacy of PHT in a chronic mTLE rodent model. The proposed method of FUS-mediated region-specific drug unbinding technique would provide a new mode of targeted drug delivery without increasing the systemic dose, with potential applications in preventing aggregations of protein-based macromolecular drugs and endogenous neuroactive protein oligomers that are mediated by weak intermolecular interactions.

## Supplementary Information

Supplementary Table S1.

## Data Availability

The datasets used and/or analyzed during the current study are available from the corresponding author on reasonable request.
