# Supplementary material for: Transcranial focused ultrasound-mediated unbinding of phenytoin from plasma proteins for suppression of chronic temporal lobe epilepsy in a rodent model
Source: Sci Rep. 2023 Mar 13;13:4128. doi: 10.1038/s41598-023-31383-4 (PMC10011522; doi:10.1038/s41598-023-31383-4)
Supplement: Supplementary file 1 — Supplementary Table S1. [file 41598_2023_31383_MOESM1_ESM.pdf]

Supplementary Information

Transcranial focused ultrasound-mediated unbinding of phenytoin from plasma proteins for suppression of chronic temporal lobe epilepsy in a rodent model

Evgenii Kim[1], Hyun-Chul Kim[1,3], Jared Van Reet[1], Mark Böhlke[2], Seung-Schik Yoo[1] and Wonhye Lee[1,*]

[1] Department of Radiology, Brigham and Women's Hospital, Harvard Medical School, Boston, MA

[2] Massachusetts College of Pharmacy and Health Sciences University, Boston, MA

[3] Department of Artificial Intelligence, Kyungpook National University, Daegu, South Korea

Supplementary Table

Supplementary Table S1. Acoustic parameter sets used for *in vitro* equilibrium dialysis.

Supplementary Table

Supplementary Table S1. Acoustic parameter sets used for *in vitro* equilibrium dialysis. DC: duty cycle; PD: pulse duration; PRF: pulse repetition frequency; I_{SPTA}: spatial-peak temporal-average intensity; I_{SPPA}: spatial-peak pulse-average intensity. DC = PD × PRF. I_{SPTA} = DC × I_{SPPA}.

Set	DC [%]	PD [ms]	PRF [Hz]	I_{SPTA} [W/cm^2]	I_{SPPA} [W/cm^2]
1	25	50	5.0	1.25	5.0
2	25	75	3.3	1.25	5.0
3	25	100	2.5	1.25	5.0
4	50	50	10.0	2.50	5.0
5	50	75	6.7	2.50	5.0
6	50	100	5.0	2.50	5.0
